# Acceptability, engagement, and preliminary efficacy of a college human physiology course with integrated mindfulness practice to support student wellbeing

**DOI:** 10.3389/fpsyg.2024.1365778

**Published:** 2024-08-14

**Authors:** Zhuoya Zhang, Brother Chân Pháp Lưu, Diane Gilbert-Diamond

**Affiliations:** ^1^Department of Epidemiology, Geisel School of Medicine at Dartmouth, Hanover, NH, United States; ^2^Deer Park Monastery, Escondido, CA, United States; ^3^Department of Medicine, Geisel School of Medicine at Dartmouth, Hanover, NH, United States; ^4^Department of Pediatrics, Geisel School of Medicine at Dartmouth, Hanover, NH, United States

**Keywords:** mindfulness, young adults, college student, health, wellbeing

## Abstract

**Objective:**

To evaluate the acceptability of and engagement with an undergraduate human physiology course embedded with mindfulness practice. To assess its preliminary efficacy on student mindfulness and wellbeing.

**Methods:**

Students (*N* = 36, 17% freshman, 33% sophomore, 22% junior, and 28% senior) answered online surveys at course completion. Primary outcomes were course ratings, assignment and assessment completion rates, minutes, types of mindfulness practice, changes in trait mindfulness (Mindful Attention Awareness Scale, MAAS), and self-reported wellbeing outcomes. We ran Chi-square goodness of fit tests and paired Wilcoxon signed-rank tests to decide if the outcomes differed significantly post-class. We tested the dose-response relation between mindfulness practice time and trait mindfulness and whether the out-of-class practice time was consistent across the weeks with generalized linear mixed-effect models.

**Results:**

All participants reported finding the course enjoyable and that they would recommend it to their friends. They practiced for an average of 66 (SD = 27) min per week in the class and 112 (SD = 59) min on their own. The most common out-of-class practices were mindful movements, sitting meditation, and breathing. Per self-reports, the course increased student understanding of specific mindfulness practices and appreciation for their body. It improved wellbeing and trait mindfulness (MAAS mean within-person change = 1.2, SD = 0.8, *p* < 0.00001). We found no does-response relation between practice time and trait mindfulness.

**Conclusions:**

This pilot study supports that incorporating mindfulness practice into college-level courses may promote student wellbeing and such approaches warrant further investigation.

## 1 Introduction

Many college students struggle with maintaining physical wellbeing (Lund et al., 2010; Ball et al., 2018; Amore et al., 2019). Poor and short sleep is prevalent in this population (Hershner and Chervin, 2014), and some report using prescription or recreational psychoactive drugs to overcome sleepiness during the day (Lund et al., 2010). Students also report struggling with healthful eating (Amore et al., 2019) and experiencing episodic overeating and low intake of fruits and vegetables (Dartmouth Office of Institutional Research, 2018). Physical inactivity is another concern, with students spending about 9.8 h a day being sedentary (Castro et al., 2020) and reporting that they lack the time, energy, and willpower to exercise (Ball et al., 2018). Elements foundational to physical health are often not met in the college population.

Mental health is another challenge. Sixty percent of U.S. college students had at least one mental health condition in 2021 (Lipson et al., 2022). Students report bearing chronic stress, academically from the intense workload and peer competition (McIntyre et al., 2018; Barbayannis et al., 2022) and non-academically from challenges in the realms of finance, career paths, and relationships (Karyotaki et al., 2020). Biologically, college students' brains are still developing the capacity to handle stress as the prefrontal cortex, the region for emotion regulation, is still growing at this life stage (Gogtay et al., 2004). College students are thus more vulnerable to unhealthy and potentially dangerous behaviors, for instance, using substances, including alcohol and drugs, as a coping mechanism for stress (Nelson et al., 2008). Finding effective means to support college students' emotional health is thus a vital public health priority (Office of the Surgeon General, 2021).

Evidence suggests that mindfulness training may enhance college students' wellbeing. Meta-analyses found small to moderate effect sizes of mindfulness-based interventions (MBIs), such as the Mindfulness-Based Stress Reduction program (MBSR; Kabat-Zinn, 2009), on university student mental health outcomes, including distress and anxiety (Dawson et al., 2020; Chen et al., 2023; Gong et al., 2023; Johnson et al., 2023; Reangsing et al., 2023). Mindfulness may also improve students' physical health. Loucks et al. found that a composite score of validated physical, mental, and social health instruments (Young Adult Health Summary Score) improved in participants who completed a 9-week mindfulness course (Mindfulness-Based College Program, MB-College, an adapted MBSR course). In contrast, the score worsened in those who received usual care over the academic semester (Cohen's *d* of 0.48, *p* = 0.004 via intention to treat analysis; Loucks et al., 2021).

Common mindfulness exercises in mindfulness-based interventions include mindful breathing, mindful movement, and deep relaxation practices such as a body scan. During these isolated practices (also known as formal practice in some mindfulness programs), the practitioners set aside time to engage in mindfulness meditation of the body and the mind. In mindful breathing exercises, practitioners become aware of their breathing and use it as an anchor to bring them back to the present moment (Kabat-Zinn, 2009; Williams and Penman, 2011). Several randomized control trials (RCTs) show that college students who practiced mindful breathing daily experienced less general anxiety (Chen et al., 2013), learning-related anxiety (Cho et al., 2016; Britt et al., 2018), depression, and perceived stress (Komariah et al., 2022). Mindful movement, such as yoga and walking meditation, helps individuals to slow down and connect with the body (O'Donovan, 2015). As one mindfully walks, one may experience a sense of wonder and connection with one's body, the people with whom they walk, and their surrounding environment (Hanh and Weare, 2017). Research shows that college students reported higher state and trait mindfulness, better mood, and lower state anxiety and stress after completing mindful walking interventions (Ma et al., 2023; Burdick and Camhi, 2024). Deep relaxation exercises, such as the body scan, invite practitioners to sequentially direct their attention to different body regions with compassionate, accepting, and curious awareness (Williams and Penman, 2011). Experimental studies on college students show that a brief body scan exercise improved students' state mindfulness (Ahmadyar et al., 2024) and lowered stress (Desai et al., 2021). A meta-analysis of 15 RCTs showed that adults who engaged in body scan practice had increased mindfulness (Hedge's *g* = 0.268, 95% CI: 0.032, 0.504, *p* < 0.05) compared to passive controls (Gan et al., 2022).

Mindfulness practice can also occur when people bring mindful awareness into routine tasks and pay attention to the process (Hanh, 1992). These integrated mindfulness practices (also known as informal practices or home practices), do not require the practitioner to set aside distinct practice time and can be carried out “off the meditation cushion” (Kabat-Zinn, 2009). For instance, one can mindfully brush their teeth. They can pay careful attention to the rich sensory experience: the water temperature, the sensation of bubbles in their mouth, the scent and taste of the toothpaste, and the movements of their hand (Williams and Penman, 2011). In an experimental study, college students (*N* = 26) who were instructed to wash their dishes for ~ 6 min mindfully reported increases in state mindfulness and positive affect (i.e., feeling inspired) and a decrease in negative affect (i.e., feeling fear) afterward, compared to those who washed dishes following regular descriptive guidelines (*N* = 25; Hanley et al., 2015). Weaving mindfulness into various everyday activities can help students diversify their mindfulness practice routine (Galante et al., 2021), while enhancing their mindfulness and psychological wellbeing (Kakoschke et al., 2021). As college students commonly report not having time for isolated practices (Sears et al., 2011; Bamber and Schneider, 2022), integrated practices may be easier to fit into their schedule (Nardi et al., 2022). The commonality and secular nature of these activities may also make integrated practices more accessible to college students.

Existing mindfulness-based interventions are efficacious but often encounter challenges with attrition and compliance (Bamber and Schneider, 2022; Nardi et al., 2022). In a study where 28 students enrolled in an 8-week adapted MBSR course in the UK, 39% discontinued before completion (Lynch et al., 2018). In the MB-College study (*n* = 47), 18% of the college students in the intervention group withdrew before week 9 (Loucks et al., 2021). A review of qualitative studies with college students revealed that time hinders many students from fully committing to MBIs (Bamber and Schneider, 2022). For instance, in MBSR, besides attending eight 2.5-h weekly sessions and a 5-h retreat, students have daily homework of 45 min of mindfulness practice (Kabat-Zinn, 2009). College student participants reported finding it hard to fulfill that requirement, leaving them guilty and discouraged (Bamber and Schneider, 2022). Creative approaches are thus needed to make mindfulness training more accessible to the college population.

Integrating mindfulness training into college life, such as during classes, may reduce participation burden and encourage student engagement. We thus piloted a “Mindful Physiology” course at Dartmouth College, where mindfulness training was woven into an undergraduate physiology curriculum, intending to enrich students' experience of learning biology through mindfulness practice. During the didactic lectures on physiology, students also learned mindfulness techniques to increase their understanding of and appreciation for the biological mechanisms in and around their bodies that benefit their wellbeing. In class, students were guided by the lecturer to contemplate their own physiology and to observe how mindfulness practice impacts their physiology and sense of wellbeing. They were also encouraged to practice mindfulness outside class for at least 15 min weekly. To our knowledge, this course presents a novel approach to teaching and practicing mindfulness techniques in the context of an undergraduate physiology course.

This study aims to evaluate a human physiology course with integrated mindfulness practice in terms of student acceptability measured via course ratings; student engagement measured via attendance, assignment and assessment completion, and type and duration of mindfulness practice; and preliminary efficacy on improving trait mindfulness and health outcomes measured via surveys. We hypothesized that the course would be acceptable, students would have high levels of course engagement, and students would report positive changes in trait mindfulness and wellbeing post-class.

## 2 Methods

### 2.1 Study design

This is a secondary analysis of self-report data collected from participants who completed an undergraduate-level biology course with integrated mindfulness practice (Biology 3: Mindful Physiology). Assessment was conducted within 5 weeks after course completion (on average, 8 days post-course completion).

### 2.2 Study sample

Students enrolled in Biology 3: Mindful Physiology were invited to participate in this study at the end of the course term (late May 2023) via e-mail. Participants were included if they were (a) enrolled in Biology 3: Mindful Physiology in the Spring 2023 academic term and (b) able to read, write, and speak in English. Of the 48 students enrolled in the class, 75% (*N* = 36) provided written consent to participate. They reviewed the study information sheet, informing them that participation would not influence their course grades and that the instructor would not know their enrollment status until course grades were finalized. Dartmouth College Committee for the Protection of Human Subjects approved the study protocol (Protocol No. 00032736).

### 2.3 Course description

Mindful Physiology (BIOL 3) was an undergraduate-level biology course open to students of all majors at Dartmouth College (New Hampshire, USA; Supplementary material 1). The course met 17 times over the 9.5-week Spring 2023 term (3/28/2023–5/30/2023). Each 110-min class session included ~20 min of group mindfulness practice. The course was taught by Professor Diane Gilbert-Diamond, ScD, a lay practitioner who practices mindfulness in the Plum Village Tradition of Thích Nhất Hạnh. This course aimed to foster a greater understanding of and appreciation for the biological mechanisms inside and around our bodies, promote scientific literacy and increase students' familiarity and capacity for mindfulness practice.

#### 2.3.1 Course content

The course introduced foundational concepts in human physiology, including but not limited to the functions of the renal, respiratory, digestive, musculoskeletal, and nervous systems. Course reading included *Hole's Human Anatomy and Physiology* (Welsh and Prentice-Craver, 2021) and *Peace is Every Step* by Hanh (1992). Readers can find the course outline in Supplementary material 1.

#### 2.3.2 Mindfulness practice

Each class had interspersed ~ 20 min of group mindfulness practice relevant to the course topic, i.e., mindful breathing, mindful eating, singing meditation, mindful movements, and deep relaxation. The course instructor (D.G.D.) led the group mindfulness practice in class based on the scripts and guidance created by the Plum Village.

Students were also encouraged to practice mindfulness outside class every day for 15 min or longer. As part of the class assignment, students logged their duration and type of mindfulness practice at least 5 days a week. To encourage candid reflection, students received full credit for completing logs, even if < 15 min of practice were reported. Students were provided with a document on resources for guided mindfulness practices, including those found on mindfulness apps such as Headspace and Plum Village, and offered in-person and online through the Dartmouth Student Wellness Center. In line with the Plum Village Tradition, students were also instructed to practice mindfulness through integrated practice in class and through course reading *Peace is Every Step* by Hanh (1992). Students could choose to practice outside class by incorporating mindfulness into everyday activities, such as brushing their teeth, walking to class, and drinking beverages with mindful attention.

In place of the term's 5th week's class meetings, students participated in a retreat with senior Dharma teachers from Thích Nhất Hạnh's Plum Village Community of Engaged Buddhism's Deer Park Monastery (2015). Students could attend the 4-h retreat, the 2-day (14-h) retreat, or both. The retreat was included in the course, to allow students to practice mindfulness techniques in a prolonged period and receive guidance from the Dharma teachers. In lieu of the retreat if students had a scheduling conflict, they were asked to complete 4 additional hours of group mindfulness practice. Class and retreat attendance counted toward a class participation grade.

#### 2.3.3 Class assignments

Class assignments included six quizzes, three problem sets, class participation including mindfulness practice logs, and weekly written reflections (Supplementary material 1). Students completed the quizzes in class. The first five quizzes covered the renal, respiratory, digestive, musculoskeletal, and nervous systems; the last quiz was cumulative. The three problem sets covered the scientific research methods content taught in the class. Further, each week, they submitted a written reflection of at least 250 words about their experiences with mindfulness practices and/or the biological concepts.

### 2.4 Outcome assessment

Students completed online questionnaires remotely after course completion (late May to mid-July 2023) administered via RedCap, an electronic data management platform hosted at Dartmouth College (Harris et al., 2009, 2019). We extracted class attendance, assignment and assessment completion, and mindfulness practice logs from class records for consented students.

#### 2.4.1 Course acceptability and effectiveness

Course acceptability and effectiveness were assessed via the post-class online survey. The survey contained 16 items, evaluating acceptability (five items, i.e., “I found the course enjoyable”), knowledge gain (four items, i.e., “I increased my understanding of my own physiology”), and self-efficacy (seven items, i.e., “I gained confidence in my ability to practice mindfulness”). Items were adapted from theoretical frameworks for evaluating intervention acceptability (Sekhon et al., 2017) and existing questions that measure class satisfaction and effectiveness (Douglas et al., 2006; Dartmouth Center for the Advancement of Learning, 2016; Bieleke et al., 2021; UC Berkeley Center for Teaching Learning, 2024). Students rated each question on a five-point Likert scale (1: strongly disagree, 2: disagree, 3: neutral, 4: agree, and 5: strongly agree). The questionnaire demonstrated good internal consistency in our sample (Cronbach's α = 0.88). Subscales showed moderate to high internal consistency (acceptability α = 0.62; knowledge gain α = 0.72; self-efficacy α = 0.86).

#### 2.4.2 Class engagement

We extracted class and retreat attendance data and the number of assignments and assessments completed from the course gradebook. Participants logged their mindfulness practice during the term five times or more a week via Google Forms. We summarized the types and durations of out-of-class exercises from the logs. Two authors (D.G.D. and Z.Z.) pre-determined the criteria for categorizing the out-of-class practice. They are (1) mindful movements, e.g., mindful walks, (2) sitting meditation, (3) mindful breathing, (4) mindful eating and drinking, (5) deep relaxation, e.g., body scan, (6) mindful art and music, e.g., singing meditation, (7) journaling, (8) listening to or reading materials from spiritual leaders, (9) mindful socializing, (10) mindful housekeeping, e.g., doing dishes mindfully, (11) mindful personal hygiene, e.g., brushing teeth mindfully, (12) other, e.g., mindful studying. One author (Z.Z.) reviewed the log entries, classified them into one of the 12 categories, and then further classified each entry as self-directed vs. guided by others, nature involved vs. not, and integrated (i.e., mindfulness embedded into daily tasks) vs. isolated practice. The two authors (D.G.D and Z.Z.) reviewed log data, resolved discrepancies through discussion and agreed on the final categorizations.

The total out-of-class practice time was the sum of minutes practiced reported in the logs. The in-class practice time was based on class attendance data (the number of sessions attended ^*^ 20 min/session). Any absence of in-class attendance or home practice was counted as 0 min of in-class or out-of-class practice, respectively. We collected the participation status of the in-person on-campus retreat (4-h retreat, 16-h retreat, or both) from the event registration records and confirmed it with each participant. Finally, we summed the minutes of in-class and out-of-class practice to compute the total minutes practiced during the term.

#### 2.4.3 Self-reported changes in trait mindfulness

After completing the course, participants completed the Mindful Attention Awareness Scale (MAAS) once to report their current level of dispositional mindfulness and a second time to retrospectively assess their level before taking the course (Brown and Ryan, 2003). The MAAS is a validated self-report instrument on trait mindfulness, i.e., one's attention to and awareness of the present moment. Participants rate how often they engage in behaviors of inattention or mindlessness (15 items, i.e., “I rush through activities without being really attentive to them”). The responses are anchored from 1 (almost always) to 6 (never) and averaged across all items for a single score. Possible scores range from 1 to 6. Higher scores reflect greater dispositional mindfulness. We did not include the item on mindful driving in our analysis, as 20% (*n* = 7) of our student participants reported that this item was irrelevant to them. The MAAS scale shows a high internal consistency in the original study (α ≥ 0.82) and in our sample (α = 0.82; Brown and Ryan, 2003). We calculated a MAAS change score with the difference between the score self-reported for pre- and post-class.

#### 2.4.4 Self-reported changes in wellbeing

Students self-assessed changes in wellbeing and related behaviors via the post-class questionnaire. Items were developed and adapted from prior research (Greaney et al., 2021). Students were invited to rate 12 statements to reflect on how the course contributed to changes in their physical (four items, e.g., ability to get quality sleep), emotional (five items, e.g., ability to manage academic stressors), and social wellbeing (three items, e.g., sense of community in the classroom). Students also assessed a statement on changes in emotional eating. Items were anchored on a five-point scale from 1: very negative changes to 5: very positive changes. The questionnaire demonstrated high internal consistency in our sample (overall α = 0.89; subscale physical health α = 0.73; emotional health α = 0.83; social health α = 0.75).

### 2.5 Covariates

We extracted participant class years (i.e., freshman, sophomore, junior, and senior) from the course registration system. Students self-reported their gender identities in the course survey administered at the course's first session.

### 2.6 Analytical approaches

Summary statistics were computed to understand the distribution of the primary outcomes and covariates. Next, we examined if the out-of-class practice time changed over the term with a linear mixed-effect model with a fixed effect by time and random intercepts by the participant. Chi-squared goodness of fit tests against a uniform distribution were performed to decide if the observed responses on the other survey items were likely due to chance. Paired Wilcoxon signed-rank tests were performed to assess whether dispositional mindfulness (MAAS item and total scores) self-reported for pre- and post-class differed significantly. Cronbach's α was computed to evaluate the internal consistency of the self-report instruments in our sample. Lastly, we examined the dose-response relation between the duration of mindfulness practice and dispositional mindfulness via linear mixed-effect models accounting for the time trend and repeated sampling. We performed the analyses at a two-sided alpha level of 0.05 and created the figures in RStudio software version 2023.06.2+561 (R Core Team, 2022).

## 3 Results

### 3.1 Student characteristics

Thirty-six undergraduate students participated in our study. Seventeen percent were 1st-year students, 33% were sophomores, 22% were juniors, and 28% were seniors (Table 1). Fifty-three percent identified with the “she/her” pronouns, 42% with “he/him,” and 3% with “she/her/they/them.”

**Table 1:** Characteristics of the class and mindfulness practice (*N* = 36).

	***N* (%) or mean ±SD**
**Class year**	
Freshman	6 (17)
Sophomore	12 (33)
Junior	8 (22)
Senior	10 (28)
**Self-identified gender pronouns**	
She/her	19 (53)
He/him	15 (42)
She/her/they/them	1 (3)
Missing	1 (3)
**Class attendance (sessions out of 17 offered)**	
17	11 (31)
16	12 (33)
15	5 (14)
11–14	8 (22)
Completion of all six quizzes	36 (100)
Completion of all three problem sets	35 (97)
**Mindfulness practice duration**	
Total (hours/term)^a^	30 ± 12
In-class (minutes/week)^b^	66 ± 27
Out-of-class (minutes/week)	112 ± 59
**Number of practice types explored outside class**	6 ± 2
**Mindfulness retreat attendance**	
Attended a 4-h retreat	28 (78)
Attended a 2-day (14-h) retreat	4 (11)
Attended both	2 (6)
Attended 4 h of group mindfulness events in lieu of retreat	2 (6)

^a^The sum of in-class and out-of-class practice time and retreat duration.

^b^The number of class sessions attended ^*^ 20 min/session.

### 3.2 Course acceptability and effectiveness

**Figure 1:**
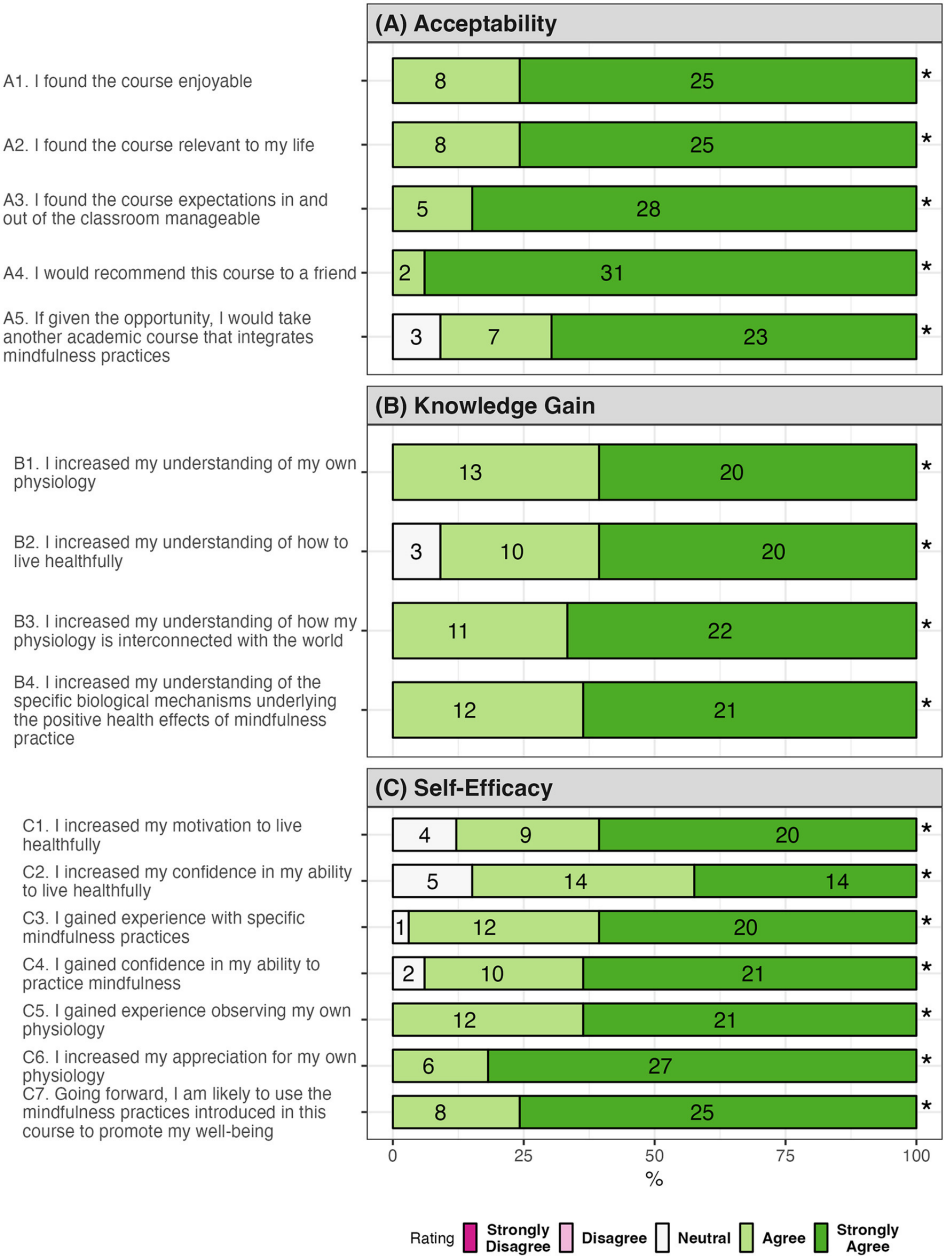
Course acceptability and attainment of learning objectives (*N* = 33). ^*^p < 0.00001 from Chi-squared goodness of fit test against a uniform distribution. **(A)** Acceptability. **(B)** Knowledge gain. **(C)** Self-efficacy.

The acceptance of the course was high. All participants found the course enjoyable and relevant to their lives (Figure 1A). All agreed that the class increased their understanding of their physiology and its interconnectedness to the world, and they would recommend the class to a friend (Figure 1, items B1, B3, and A4). Ninety-one percent expressed wanting to take another academic course incorporating mindfulness practice (Figure 1, item A5).

All agreed that the course enhanced their knowledge about the biological mechanisms underlying the positive health impacts of mindfulness practice (Figure 1B). Many believed that that course had increased their appreciation for their bodies (100%), knowledge of how to live healthfully (91%), and motivation to live healthfully (88%; Figures 1B, C).

### 3.3 Class engagement

Participants engaged well with the course content throughout the term. All completed the six in-class assessments, and 97% completed all three problem sets (Table 1). Of the 17 class sessions offered, 64% attended at least 16 sessions, 14% attended 15, and 22% attended between 11 and 14 sessions.

Participants practiced mindfulness for an average of 30 h (SD = 12) during the term (Table 1). They practiced weekly in class for an average of 66 min (SD = 27). Seventy-eight percent attended the 4-h retreat, 11% attended the 2-day (14-h) retreat, and 6% attended both. Two students participated in alternate activities due to scheduling conflicts with the retreat.

**Figure 2:**
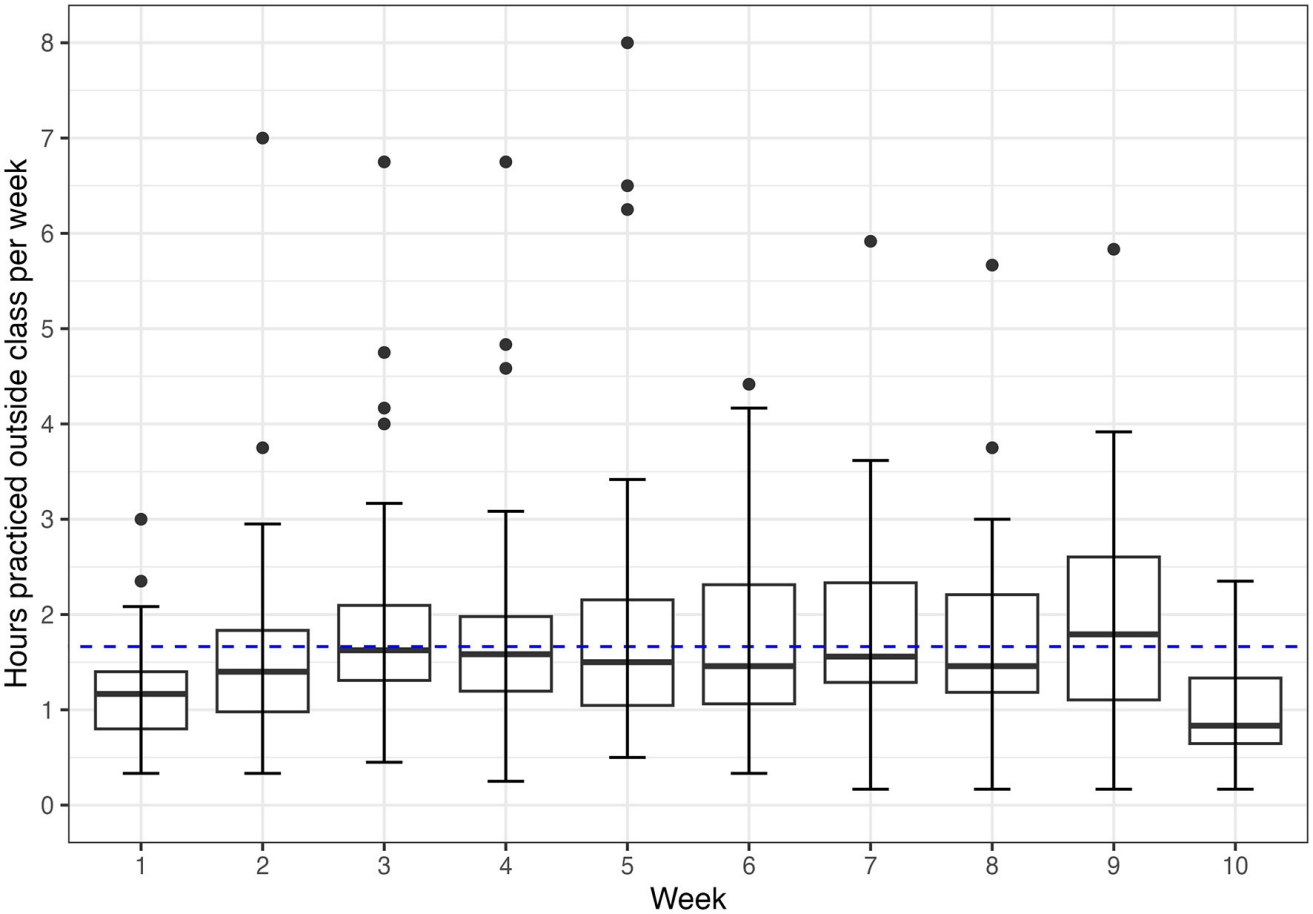
Distribution of student out-of-class mindfulness practice time over the academic term (*N* = 36). The blue dash line shows the sample average weekly practice hours (1.7 h). The box covers the range between 25 and 75th percentiles, with the line in the middle showing the median. The top and bottom whiskers indicate the minimum and maximum values, excluding the outliers (plotted as the dots).

Outside of class, participants practiced mindfulness for an average of 20 min (SD = 17) per day (Table 2). The out-of-class practice time was stable throughout the course (*p* = 0.60 for the time fixed effect coefficient in a linear mixed-effect model, Figure 2). They explored, on average, 6 (SD = 2) types of mindfulness exercises on their own (Table 1). Mindful movement was the most common out-of-class practice in this sample, and 94% of the participants described practicing self-led mindful movement activities, e.g., mindful walks and runs (Table 2). Sitting meditation was the second most common practice, and 42% of the participants described practicing with an app. Participants also described practicing mindfulness through breathing (*N* = 27), deep relaxation (*N* = 22), journaling (*N* = 13), and art and music (*N* = 17).

**Table 2:** Summary of self-reported out-of-class mindfulness practice (*N* = 36).

**Category**	**Examples**	***N* (%)**	**Minutes/Session**	
			**Mean** ±**SD**	**Median (IQR)**
Overall		36	20 ± 17	15 (11, 20)
*Nature-involved*	Mindfully having lunch outside	27 (75%)	22 ± 17	15 (15, 30)
*Mindfulness integrated into everyday activities*	Making coffee mindfully	34 (94%)	13 ± 8	12 (10, 15)
Sitting meditations		35 (97%)	20 ± 14	15 (15, 20)
*App-delivered*	Plum Village app, Headspace, Calm, or YouTube	15 (42%)	15 ± 78	13 (15, 15)
Mindful movement		34 (94%)	22 ± 17	15 (15, 25)
*Structured*	Instructor-led yoga class	7 (19%)	34 ± 29	25 (20, 45)
*Self-led*	Mindful walks, runs, bike rides	34 (94%)	21± 16	15 (15, 20)
Mindful breathing	Box breathing, paying attention to the breath	27 (75%)	13 ± 7	14 (10, 15)
Mindful eating and drinking	Drinking tea mindfully	27 (75%)	15 ± 12	15 (5, 20)
Deep relaxation	Body scans, lying on the grass	22 (61%)	18 ± 12	15 (10, 20)
Mindful art and music		17 (47%)	31 ± 31	15 (10, 45)
*Creating art*	Writing poems, playing instruments, crafting	2 (6%)	28 ± 23	22.5 (13, 38)
*Engaging with art*	Appreciating artwork in a museum, reading, listening to music	12 (33%)	38 ± 34	25 (10, 60)
*Singing*	Chanting, singing meditation	3 (8%)	10 ± 7	7.5 (5, 15)
Journaling		13 (36%)	20 ± 13	15 (13, 20)
Mindful personal hygiene	Brushing teeth mindfully	10 (28%)	16 ± 6	15 (14, 20)
Mindful socializing	Talking to friends and teachers mindfully	10 (28%)	33 ± 32	30 (15, 45)
Mindful housekeeping	Washing dishes mindfully	9 (25%)	27 ± 24	20 (15, 30)
Engaging with materials from spiritual leaders	Listening to Dharma talks and mindfulness podcasts, reading books about mindfulness.	8 (22%)	36 ± 45	19 (15, 30)
Other	Mindful studying, driving	7 (19%)	56 ± 51	40 (15, 90)

Examples are excerpts from student practice logs.

N, the number of students who reported engaging with that practice at least once during the course term; SD, standard deviation; IQR, interquartile range, the range between the 25 and 75th percentiles.

In line with the philosophy of mindful living encouraged in the class reading *Peace is Every Step* (Hanh, 1992), many participants incorporated mindfulness into daily tasks. Examples are mindful eating and drinking (*N* = 27, 75%), mindfully socializing with others (*N* = 10, 28%), and housekeeping (*N* = 9, 25%; Table 2). Seventy-five percent also described practicing mindfulness in nature.

### 3.4 Changes in trait mindfulness

**Figure 3:**
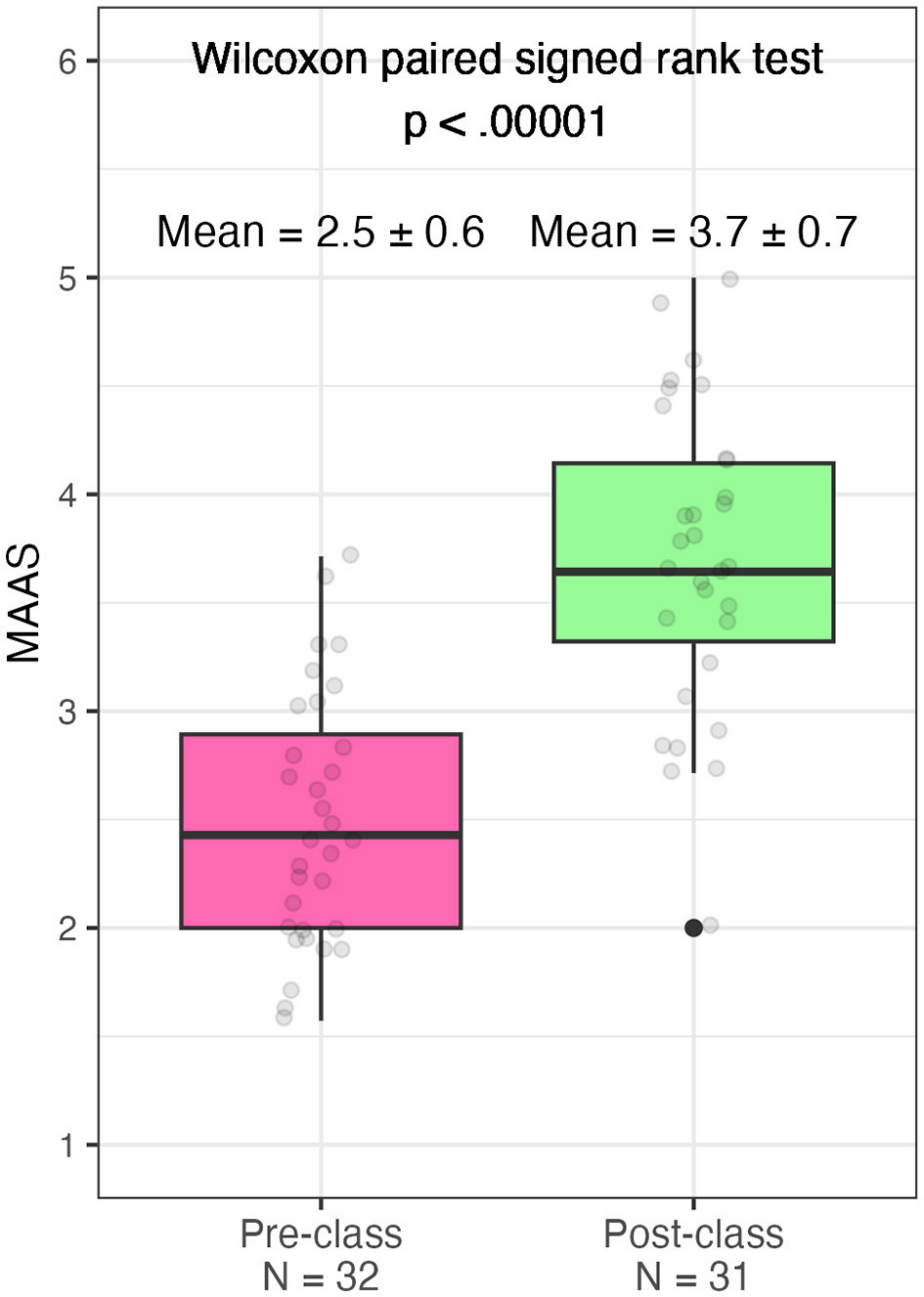
Distribution of Mindful Attention Awareness Scale (MAAS) scores reported for before and after class. The box covers the range between 25 and 75th percentiles, and the line in the middle represents the median score. The top and bottom whiskers indicate the minimum and maximum values, excluding the outliers. Each dot presents a student's score.

Participants reported that their attention to the present moment and capacity for mindfulness improved after taking the class, with an average of 1.2 unit within-person increase in the MAAS score (SD = 0.8, *p* = 1.82 × 10^−6^ from paired Wilcoxon signed rank exact test, *N* = 30; Figure 3).

**Figure 4:**
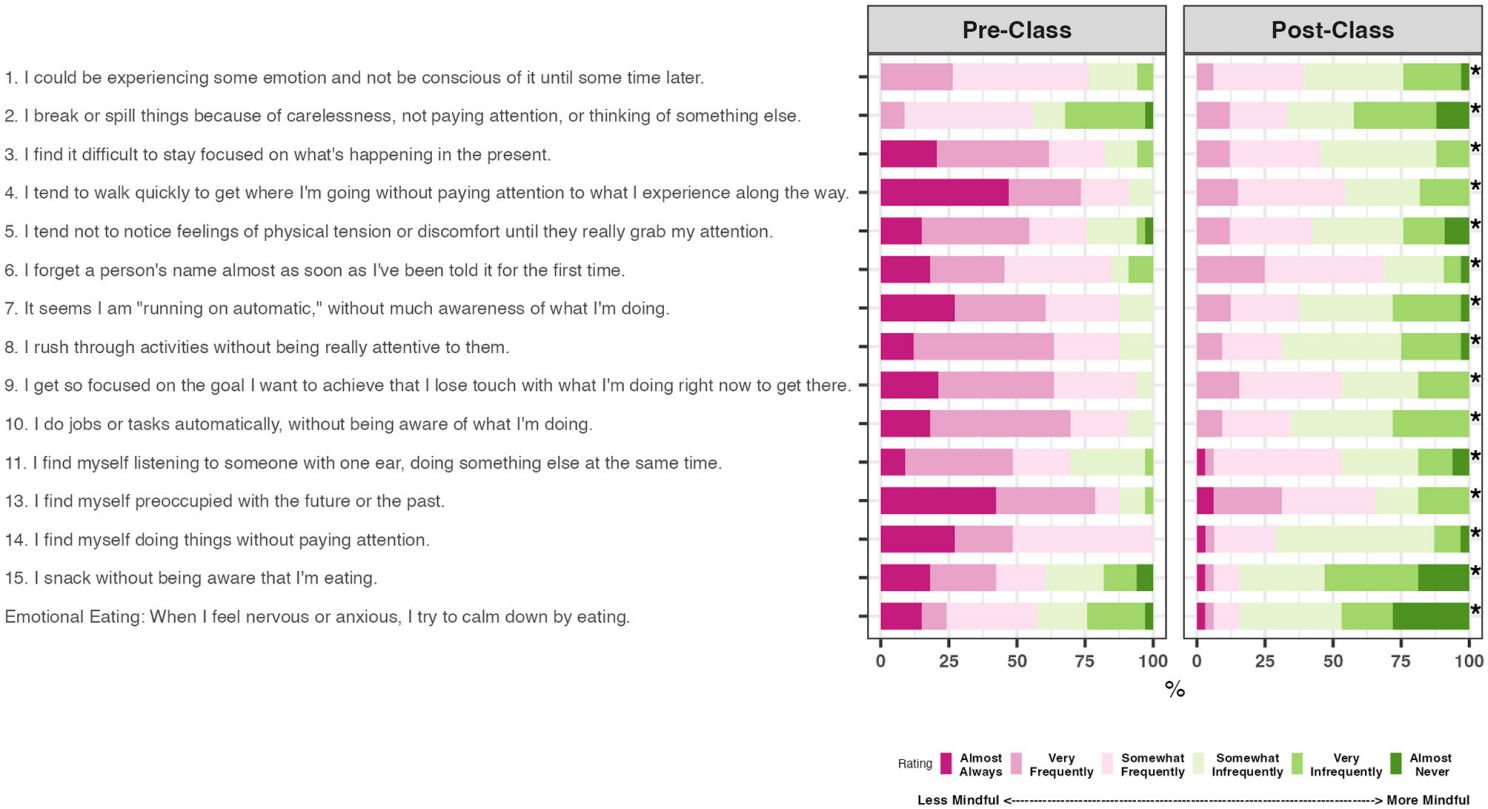
Responses on the Mindful Attention Awareness Scale (MAAS) and emotional eating behavior reported for before and after class (*N* = 31). Item 12 on mindful driving in the original MAAS questionnaire was not included, as 20% of this college student sample reported this item was not applicable. *p < 0.00001 via paired Wilcoxon signed-rank tests.

We observed a statistically significant increase in each MAAS item score (*p* < 0.00001 from paired Wilcoxon signed rank exact test; Figure 4). Many reported a drop in their tendency to walk mindlessly and operate on auto-pilot (Figure 4, items 4 and 7). Participants also found themselves less likely to get caught up in their thoughts about the past or the future (Figure 4, item 13). We did not observe an association between total hours practiced and dispositional mindfulness evaluated via the MAAS (*p* = 0.379; Table 3).

**Table 3:** Associations between reported mindfulness practice time and trait mindfulness assessed via Mindful Attention Awareness Scale (*N* = 33).

**Independent variable**	**Unadjusted**		**Adjusting for gender and class year**	
	**β** ^**a**^	***p***	**β** ^**a**^	***p***
Total practice hours throughout the course	0.01	0.391	0.01	0.379
Minutes of in-class practice	0.00	0.456	0.00	0.456
Minutes of out-of-class practice	0.00	0.255	0.00	0.255

^a^Fixed effect coefficients from linear mixed-effect models with the time practiced as the independent variable and the MAAS scores as the dependent variable, accounting for time trend and repeated sampling, i.e., lmer [~ MAAS + time + (1|ID) + covariates] in R.

MASS, Mindful Attention Awareness Scale, with possible scores ranging from 1 to 6.

### 3.5 Changes in self-reported wellbeing

**Figure 5:**
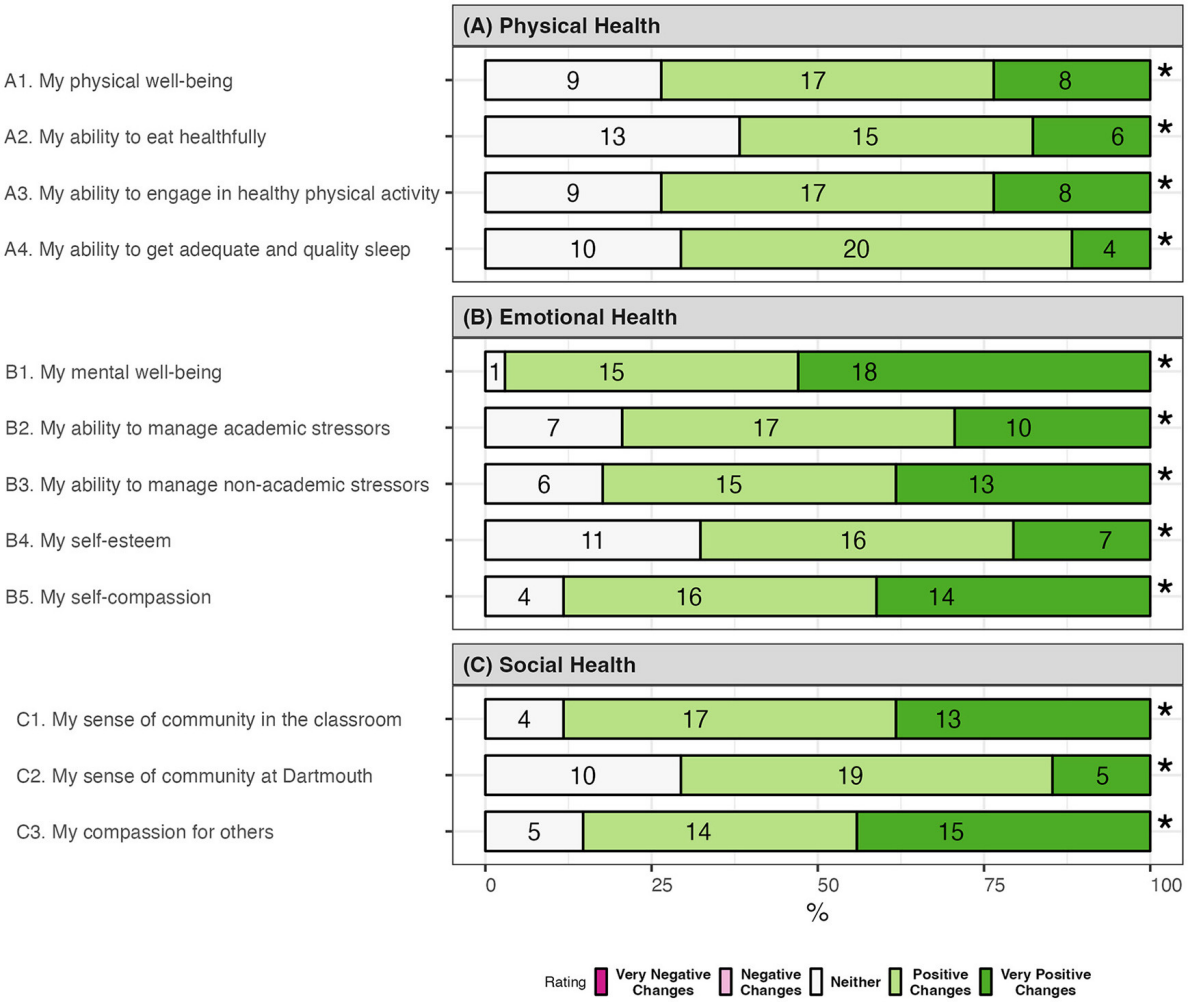
Changes in self-reported health outcomes (*N* = 34). *p < 0.00001 from Chi-squared goodness of fit test against a uniform distribution. **(A)** Physical health. **(B)** Emotional health. **(C)** Social health.

Participants answered that the course positively or very positively influenced their physical health (Figure 5A), including a greater capacity to engage in healthy physical activity (74%), get quality and adequate sleep (71%), and eat more healthfully (62%). The tendency of eating to cope with negative emotion dropped (*p* < 0.00001 from paired Wilcoxon signed-rank test, Figure 4, emotional eating).

Participants noticed positive mental and social wellbeing changes from taking this course (Figures 5B, C). Examples were more compassion toward self (88%) and for others (85%), improved self-esteem (68%), and greater capacity for managing non-academic (82%) and academic stressors (79%). Many believed the course enhanced their sense of community in the classroom (88%) and on campus (71%).

## 4 Discussion

In this Mindful Physiology course, we integrated the teaching of human physiology with related mindfulness practice. The class was well-received by undergraduate students. All primary outcomes demonstrated positive trends. Per student reports, the course was related to improvement in their trait mindfulness, familiarity of and capacity for mindfulness practice, as well as physical (i.e., sleep quality, healthful eating, and physical activity), mental (i.e., stress regulation, self-esteem, and compassion), and social wellbeing (i.e., sense of belonging). Further stringent larger-scale RCTs and qualitative studies are granted to confirm whether embedding mindfulness practice into college curricula would enhance learning and student wellbeing.

### 4.1 This human physiology course with integrated mindfulness training was acceptable to college students

The Mindful Physiology course showed high acceptability (Figure 1). Students attained academic objectives (i.e., increased understanding of human physiology) while enjoying the learning process. Unlike conventional college courses, this course invited students to practice mindfulness related to the lecture topics. Practicing inside and outside of class allowed students time to relax, cultivate joy, and reflect on and appreciate their own physiology. Research suggests that positive academic emotions, such as joy and gratitude, can increase student motivation for learning (Pekrun et al., 2017). Studies also show that learners are more willing to invest time and effort when learning is fun (Pekrun et al., 2002; Villavicencio and Bernardo, 2013). Enjoyment (i.e., positive emotion related to curiosity and interest in learning) has been shown to strongly predict academic achievement (Villavicencio and Bernardo, 2013). Future studies should investigate whether integrating mindfulness into curricula fosters positive academic emotions and benefits learning outcomes.

Participants engaged well with the course, as indicated by their class attendance, assignment, and assessment completion rates (Table 1) and consistent engagement with out-of-class mindfulness practice (Figure 2). The vast majority attended the mindfulness retreat(s) and practiced mindfulness inside and outside the class (Table 1). A qualitative review suggests that the course's approach of offering students options in the type and duration of their out-of-class practice was conducive to participant engagement with mindfulness training (Bamber and Schneider, 2022). The flexibility and autonomy potentially allowed students to fit mindfulness training into daily routines and increase motivation to practice (Table 2). Additionally, the preference for self-directed or group practice varied, with more students favoring self-directed activities. For example, participants reported engaging in more self-directed mindful walks than structured mindful movement classes (e.g., instructor-led yoga classes), and more sitting meditations guided by mobile apps than in-person at the school's wellness center (Table 2). While we did not interrogate why students chose various activities, we hypothesize that students may prefer the scheduling flexibility of self-directed activities.

### 4.2 Mindfulness practice embedded in daily activities and nature may be more accessible to college students

Students resonated with practicing mindfulness in everyday activities (integrated practice, e.g., cleaning mindfully and brushing their teeth mindfully (Table 2). Introducing students to various ways to practice may make mindfulness more accessible and sustainable. Several studies conducted among college students report that weaving mindfulness into daily tasks, such as dishwashing, may increase state mindfulness and positive emotion (Hanley et al., 2015; Hindman et al., 2015; Cebolla et al., 2017). Integrated practice may also reduce the anxiety and guilt felt by students when they cannot find time for isolated sitting meditations (Bamber and Schneider, 2022). A follow-up study of an 8-week college mindfulness program found integrated practice sustainable; a year after, most students reported practicing mindfulness in everyday activities, e.g., mindful eating and mindful walks, while very few continued practicing isolated meditation practices (Galante et al., 2021). Importantly, Galante et al. found a dose-response benefit regardless of whether the practice was integrated or isolated. Introducing options and the flexibility to create one's own practice routine may encourage more sustainable and enjoyable mindfulness practice for college students.

Our participants also favored nature-involved practices, such as mindful walking, running, or eating outdoors (Table 2). A review suggests that contact with nature (“green time”), even as briefly as 10 min of sitting or walking in a natural setting, is linked to improved psychological wellbeing markers in university students (Meredith et al., 2020). Studies also indicate that contact with nature is linked to greater happiness (Mayer et al., 2009) and lower anxiety (Mackay and Neill, 2010). Mindful attention when interacting with nature may augment its beneficial effects; an experiment by Nisbet et al. found that college students who walked outdoors for 20 min while being guided with mindfulness instructions reported a stronger sense of nature connectedness and positive emotions than those who walked indoors or outdoors without mindfulness instruction (Nisbet et al., 2019). Colleges can consider further incorporating nature along with mindfulness into college courses and events, increasing student access to natural space and encouraging “green time” to nourish students' emotional health.

### 4.3 The course was associated with increased trait mindfulness

The course increased student trait mindfulness measured via the MAAS self-reported for pre- vs. post-class (Figures 3, 4). A meta-analysis of 125 studies in diverse populations (*N* = 31,679, median age = 28.3 years, 91% non-clinical participants) supports that trait mindfulness is positively associated with healthy behaviors, such as healthy eating, sleep, and exercise, with correlations ranging from 0.08 to 0.14 (Sala et al., 2020). Epidemiological studies in college populations also suggest that more mindful students tend to exhibit more healthful behaviors; they eat more nutritiously, exercise more, and spend more time on self-care (Murphy et al., 2012; Lyzwinski et al., 2019) and report less problematic alcohol use and binge eating (Bodenlos et al., 2013, 2015; Giannopoulou et al., 2020). However, the literature is mainly cross-sectional. Understanding whether trait mindfulness prospectively promotes healthy behaviors thus requires further investigation in experimental and longitudinal studies.

We did not observe an association between reported mindfulness practice time and trait mindfulness measured via the MAAS (Table 3). However, the exposure variable of practice time is limited because it does not account for practice quality and is subject to measurement error from recalls. Future research can improve this aspect by asking students to evaluate the quality of their mindfulness practice with validated instruments, e.g., the PQ-M (Del Re et al., 2013). More frequent sampling via ecological momentary assessment (EMA) may reduce some measurement errors from recalls (Moore et al., 2016; Grégoire and Doucerain, 2022).

While we did not further interrogate, we hypothesize that other course components, such as course readings, physiology lectures, and mindfulness retreats, may also have contributed to the increase in student dispositional mindfulness. Potentially, the combination of learning about human physiology and connecting with the body through mindfulness practice positively affects trait mindfulness. Past research also indicates that the instructors'/facilitators' teaching style may influence the students' development of mindfulness skills (Hanh and Weare, 2017; Bamber and Schneider, 2022). For instance, participants from an 8-week mindfulness program expressed that teachers who modeled and practiced mindfulness inspired them to do the same (Shonin et al., 2014). Integrating mindfulness throughout the classroom has been shown to facilitate teaching and learning, as students are more present and engage better (Schwind et al., 2017). Future research should include structured qualitative interviews with participants to elucidate which course components contribute to changes in trait mindfulness.

### 4.4 The course was associated with improved self-reported physical health

Improvement in self-reported physical wellbeing and health behaviors was also pronounced in our study. More specifically, most students reported that the course had positive or very positive changes in their ability to get adequate and quality sleep, engage in healthy physical activity, and eat healthfully (Figure 5). In line with Bandura's social cognitive theory of individual behavioral change (Bandura, 1977, 1997), these behavioral changes may have been supported by students increased understanding of the physiological benefits of these behaviors, along with their increased confidence in their ability to engage in these behaviors (Figure 1). Other studies also show that increased self-efficacy and outcome expectations may induce successful physical activity and dietary behavior changes (Young et al., 2014; Luszczynska and Schwarzer, 2020; Sebastian et al., 2021).

Positive behavioral changes in participant sleep, nutrition, and exercise may also have derived from reduced perceived stress and better stress management skills (Figure 5B). Stress and health behaviors may present a bi-directional relationship (Donald et al., 2016; Querstret et al., 2020; Dark-Freudeman et al., 2022). For instance, evidence indicates that sleep is essential in modulating emotional stress and physiological stress responses (Minkel et al., 2014; Simpson et al., 2016; Vandekerckhove and Wang, 2017). However, acute and chronic stress, when insufficiently regulated, has been shown to disrupt sleep (Lo Martire et al., 2020). Similarly, exercise has been shown to help reduce stress, but high perceived pressure has been shown to impede one's motivation to exercise (Stults-Kolehmainen and Sinha, 2014). Reducing stress is thus vital to breaking this vicious cycle. With mindfulness training, students can experience less stress and cultivate more feelings of ease and calm, which may facilitate positive health behaviors. Further research should interrogate whether reductions in stress mediate the observed improvements in health behaviors and physical health.

### 4.5 The course was associated with improved self-reported emotional and social wellbeing

Participants also reported positive changes in their self-esteem and compassion for themselves and others after taking the class (Figure 5). Prior research supports that trait mindfulness positively relates to self-esteem (i.e., one's sense of self-worth) and that mindfulness training can increase self-esteem (Pepping et al., 2013) potentially via reduced negative thinking (Frewen et al., 2008) and self-criticism (Dundas et al., 2016; Noh and Cho, 2020). With mindfulness, one can practice challenging unhelpful thought patterns and embracing self-compassion and a growth mindset instead (Saraff et al., 2020). Studies show that exercises such as loving-kindness meditation and supportive touch (e.g., hand massage), which some participants reported practicing, can increase empathy (Boellinghaus et al., 2014; Goldstein et al., 2017). Future research should explore if specific types of mindfulness practice are particularly beneficial for emotional health in college students.

The increase in the sense of community was also notable in our participants (Figure 5C). Young adults may experience more loneliness, because they are often single and live alone (Hawkley et al., 2022; Ellard et al., 2023). Studies report that attitudes of interconnectedness, acceptance (i.e., openness and receptivity of the present-moment experience), and compassion cultivated through mindfulness practice may help alleviate loneliness (Teoh et al., 2021; Xie et al., 2023) and enhance interpersonal relationships (Lindsay et al., 2019). Interestingly, 28% of our sample described practicing mindful socialization (e.g., mindfully talking and listening to a friend, connecting with their pets) in their spare time (Table 2). Further studies should use validated instruments of social connectedness (Veazie et al., 2019; Ellard et al., 2023) to more rigorously explore the effects of practicing mindfulness overall and specifically while socializing on reducing loneliness.

### 4.6 Study strengths, limitations, and future directions

This Mindful Physiology course introduced an innovative approach to teaching human physiology with incorporated mindfulness practice. We evaluated the course acceptability, engagement, and potential impacts on student wellbeing via questionnaires. Nevertheless, the pilot study has limitations, including small sample size (*N* = 36 out of 48 registered students), inadequate assessment of covariates (e.g., race/ethnicity, prior experience with meditation, health status, and major), absence of a comparison group, and absence of an assessment administered at baseline. Our study is further limited by potential bias from self-selection of the sample, potential measurement error in self-reports, and potential limited assessment on the non-judgment facet of mindfulness due to the unidimensional design of the MAAS (Baer et al., 2006; Baer, 2019). These limitations preclude us from making causal inferences and may limit our study's generalizability.

Future studies should consider conducting validated subjective questionnaires, such as the Warwick-Edinburg Student Wellbeing questionnaire (Tennant et al., 2007) and objective measurements, such as hair cortisol, heart rate, and accelerometry (Russell et al., 2012; Cain et al., 2013; Kim et al., 2018), with a pre-and post-intervention design, to assess changes in student health and behaviors. Further, qualitative studies with student participants can provide insights into the facilitators and barriers to practicing mindfulness in college life. Nonetheless, our study suggests that the course may have positively impacted students' physical and mental wellbeing and supports that larger-scale RCTs are warranted to confirm these benefits.

### 4.7 Implications for educational practices

This study and others support that incorporating mindfulness into higher education can potentially improve student wellbeing. Although our class was based on the intersection of mindfulness and biology, the concepts (e.g., interconnectedness and impermanence) and skills (e.g., focused attention) introduced and cultivated through mindfulness practice are not unique to the discipline of biology. For instance, in a chemistry class, teachers may invite students to contemplate the idea of impermanence while observing a chemical reaction. In an ecology class, students may be invited to reflect on their role and the impact of their actions on the ecosystem to understand the idea of interbeing. Teachers may lead a brief mindfulness meditation in a language class to reduce language learning anxiety (Zeilhofer and Sasao, 2022). Simply reflecting on the class content and its relevance to daily life can improve learning outcomes (Priniski et al., 2018).

Incorporating mindfulness into higher education curricula may be challenging due to the need for a professor to feel comfortable in both their subject area and mindfulness practices. For professors interested in incorporating such an approach but less experienced with mindfulness practices, collaborations with mindfulness experts to co-design the curriculum may be feasible. In this course, monastics from the Plum Village Tradition engaged with students through in-class visits and on-campus retreats. Given the limited availability of monastic mindfulness experts and the potential burden of running retreats, future research should explore whether monastic engagement and mindfulness retreat attendance were necessary for the positive changes reported by the students. As the world faces a crisis in youth's mental health, further research into integrating mindfulness into academic courses can provide the necessary evidence to support or refute the approach of incorporating mindfulness to foster positive student learning and health outcomes.

## 5 Conclusions

Mindful Physiology, a college biology course with integrated mindfulness practice, demonstrated high acceptability, student engagement, and effectiveness in this pilot study. Per student reports, the class was related to improved trait mindfulness and physical, mental, and social wellbeing. Further evidence from larger-scale, stringent experimental and qualitative studies is needed to make causal inferences. Nevertheless, this study provides preliminary evidence that incorporating mindfulness into higher education curricula may enhance students' learning experience and wellbeing.

## Data availability statement

The raw data supporting the conclusions of this article will be made available by the authors, without undue reservation.

## Ethics statement

The studies involving humans were approved by Dartmouth College Committee for the Protection of Human Subjects. The studies were conducted in accordance with the local legislation and institutional requirements. The participants provided their written informed consent to participate in this study.

## Author contributions

ZZ: Data curation, Formal analysis, Methodology, Validation, Visualization, Writing – original draft, Writing – review & editing. BL: Conceptualization, Methodology, Writing – review & editing. DG-D: Conceptualization, Data curation, Investigation, Methodology, Project administration, Resources, Software, Supervision, Validation, Visualization, Writing – review & editing.
