# Supplementary material for: Acceptability, engagement, and preliminary efficacy of a college human physiology course with integrated mindfulness practice to support student wellbeing
Source: Front Psychol. 2024 Aug 14;15:1365778. doi: 10.3389/fpsyg.2024.1365778 (PMC11350291; doi:10.3389/fpsyg.2024.1365778)
Supplement: Supplementary file 1 [file Data_Sheet_1.PDF]

Supplementary Material

Acceptability, Engagement, and Preliminary Efficacy of a College Human Physiology Course with Integrated Mindfulness Practice to Support Student Well-being

Zhuoya Zhang*, Brother Chân Pháp Lưu, Diane Gilbert-Diamond

*** Correspondence:** Zhuoya Zhang: zhuoya.zhang.gr@dartmouth.edu

File S1. Mindful Physiology Course Outline

Instructor: The course was taught by Professor Diane Gilbert-Diamond, ScD.

Class Meetings: The course met on Tuesdays and Thursdays from 3/28/2023 to 5/30/2023, with the exception of 4/25 and 4/27. Class sessions included ~90 minutes of didactic lecture on human physiology interspersed with ~20 minutes of guided mindfulness practice, except for the 3/28 and 4/20 sessions that included discussions with Plum Village monastics about mindfulness practice. Students were required to attend either the 4-hr mindfulness retreat on 4/21 or 2-day mindfulness retreat on 4/22 and 4/23.

**Dates (m/d)**	**Class Topic**
3/28	Introduction to Mindfulness
3/30	Mindful Drinking: The Importance of Water for Life, and Homeostatic Regulation of Water
4/4, 4/6, and 4/11	Mindful Breathing: Cellular Respiration, The Circulatory and Respiratory Systems
4/13	Research Methods Introduction I
4/18	Mindful Eating: The Digestive System
4/20	Question and Answer Session with Plum Village Monastics
4/21 - 4/23	Mandatory Attendance at 4-hr or 2-day retreat
4/25 and 4/27	No class meetings to return time spent at the mindfulness retreat
5/2	Mindful Eating: The Digestive System Continued
5/4 and 5/9	Mindful Walking: The Muscular System
5/11	Research Methods Introduction II
5/16, 5/18, and 5/23	Deep Relaxation: The Nervous System
5/25	Research Methods Introduction III
5/30	Class Wrap Up

Grades:

Quizzes: (60% of course grade) Students completed 6 quizzes on the physiologic content taught in the class.

Problem Sets: (10% of course grade) Students completed 3 problem sets on the scientific methods content taught in the class.

Class Participation including mindfulness practice logs: (20% of course grade) In addition to focusing during lectures, participating in discussions, and engaging in activities, students were also asked to engage in 15 minutes of daily mindfulness practice. To encourage accurate reporting, students were given credit for completing logs, even if <15 minutes of practice was reported.

Written Reflections: (10% of course grade) Each week students received credit for completing a written reflection of at least 250 words about their own experiences with their mindfulness practice and/or the biological concepts covered in the course.
